# Coexistence of protease sensitive and resistant prion protein in 129VV homozygous sporadic Creutzfeldt–Jakob disease: a case report

**DOI:** 10.1186/1752-1947-6-348

**Published:** 2012-10-11

**Authors:** Ana B Rodríguez-Martínez, Adolfo López de Munain, Isidro Ferrer, Juan J Zarranz, Begoña Atarés, Nuria T Villagra, Jose M Arteagoitia, Joseba M Garrido, Ramón A Juste

**Affiliations:** 1 Department of Animal Health, Neiker-Tecnalia, Berreaga 1, Derio, Bizkaia, 48160, Spain; 2 Neurology Service, Hospital de Donosti, San Sebastian, Spain; 3 Institute of Neuropathology, IDIBELL-Hospital Universitari de Bellvitge, Hospitalet de Llobregat, Barcelona, Spain; 4 CIBERNED, Instituto de Salud Carlos III, Barcelona, Spain; 5 Neurology Service, Hospital de Cruces, Plaza Cruces-Gurutzeta 12, Barakaldo, Bizkaia, 48902, Spain; 6 Pathology Service, Hospital de Txagorritxu, José Achótegui s/n. 01009, Vitoria-Gasteiz, Alava, Spain; 7 Department of Health and Consumption, Gobierno Vasco, Vitoria-Gasteiz, Alava, Spain

**Keywords:** Creutzfeldt–Jakob, Prion, Protease resistance, Resistant PrP, Sensitive PrP, Variably Protease Sensitive Prionopathy

## Abstract

**Introduction:**

The coexistence of different molecular types of classical protease-resistant prion protein in the same individual have been described, however, the simultaneous finding of these with the recently described protease-sensitive variant or variably protease-sensitive prionopathy has, to the best of our knowledge, not yet been reported.

**Case presentation:**

A 74-year-old Caucasian woman showed a sporadic Creutzfeldt–Jakob disease clinical phenotype with reactive depression, followed by cognitive impairment, akinetic-rigid Parkinsonism with pseudobulbar syndrome and gait impairment with motor apraxia, visuospatial disorientation, and evident frontal dysfunction features such as grasping, palmomental reflex and brisk perioral reflexes. She died at age 77.

Neuropathological findings showed: spongiform change in the patient’s cerebral cortex, striatum, thalamus and molecular layer of the cerebellum with proteinase K-sensitive synaptic-like, dot-like or target-like prion protein deposition in the cortex, thalamus and striatum; proteinase K-resistant prion protein in the same regions; and elongated plaque-like proteinase K-resistant prion protein in the molecular layer of the cerebellum. Molecular analysis of prion protein after proteinase K digestion revealed decreased signal intensity in immunoblot, a ladder-like protein pattern, and a 71% reduction of PrPSc signal relative to non-digested material. Her cerebellum showed a 2A prion protein type largely resistant to proteinase K. Genotype of polymorphism at codon 129 was valine homozygous.

**Conclusion:**

Molecular typing of prion protein along with clinical and neuropathological data revealed, to the best of our knowledge, the first case of the coexistence of different protease-sensitive prion proteins in the same patient in a rare case that did not fulfill the current clinical diagnostic criteria for either probable or possible sporadic Creutzfeldt–Jakob disease. This highlights the importance of molecular analyses of several brain regions in order to correctly diagnose rare and atypical prionopathies.

## Introduction

Prion diseases are fatal neurodegenerative disorders characterized by the accumulation of the abnormal isoform of the cellular prion protein (PrP). The main differences between the normal physiological and the abnormal pathologic protein are their physical and chemical properties, and specifically their detergent solubility, α-helix and β-sheet content in the secondary structure, and degree of resistance to protease digestion.

A novel form of sporadic prion disease, termed variably protease-sensitive prionopathy (PSPr or VPSPr), has recently been reported [1]. This form comprises, to date, a low number of cases worldwide [1-6], and in the USA it was estimated to account for 3% of sporadic Creutzfeldt–Jakob disease (sCJD) [1]. However, it must be considered that some cases might have been initially misdiagnosed as other prion disease phenotypes such as sCJD [2,5] or atypical forms of Alzheimer’s disease or Gerstmann–Sträussler–Scheinker syndrome [4], and that the real prevalence could be higher because PSPr cases may be misclassified within the group of non-Alzheimer’s dementias [1]. In this novel sCJD variant, cases with the three affected 129 polymorphism genotypes were described [4-6] in which all the distinguishing properties of higher PrP sensitivity to proteinase K (PK) degradation and ladder-like band pattern on western blots were shown.

A common observation in sCJD is the coexistence of different PrP classical PK-resistant types in the same or in distinct brain regions [7-15]. However, to the best of our knowledge, the coexistence of different resistance PrP types in the same patient with PSPr has not yet been reported [1,2,4,6].

Here, we describe the first case of sCJD 129VV with coexistence of different protease-sensitive PrP types.

## Case presentation

### Clinical history

A 74-year-old Caucasian woman had a 36-month history of initial depression and a slow progressive cognitive impairment. Past medical history reported hypertension and cataract surgery. No information about family history of dementia was recorded. Nine years ago, in the mourning for the deaths of her spouse and other relatives, she developed a depressive disorder which was considered reactive and was left untreated. During the following months she showed bradypsychia and speech impairment without behavior disorder or evident memory loss. Ten months later, while still autonomous and able to take care of her grandchildren, she went for the first time to a neurologist. The neurological examination disclosed a bradypsychia, a depressive facies and a slightly dysarthric, monotonous and dysprosodic speech. She was cooperative, oriented with respect to time and space but she followed the instructions clumsily. She was unable to write (she was low-literate) and presented gesture ideomotor apraxia that her anxiety made difficult to examine. Akinetic-rigid syndrome or fasciculations were absent. The overall examination revealed nothing but a systolic aortic murmur.

The patient’s clinical picture was interpreted as a reactive depression in addition to an isolated language disorder characterized as pseudobulbar speech that led to the presumptive diagnosis of a frontal degenerative disorder diagnosis. Neither Parkinson’s disease with cognitive impairment, nor Alzheimer’s disease was suspected at that time. Cardinal features of progressive supranuclear palsy or motor neuron disease were absent. Additional studies included a cranial MRI showing atrophy of the bilateral frontoparietal and cerebellar hemispheres, laboratory tests for B12 vitamin, folic acid and thyroid hormone levels with normal results, as well as an echocardiograph showing mild aortic insufficiency. Treatment with paroxetine (20mg/day) was initiated. After a month, a new examination revealed worsening of the language impairment. The patient’s clinical picture matched with a progressive degenerative cognitive impairment and hence the suspicion of Alzheimer’s disease or frontotemporal dementia was raised. During the following months, the disease progressed, affecting gait and causing several falls that included a broken humerus six months later. A cranial single-photon emission computed tomography performed at that time demonstrated frontal hypocaptation more prominent on the left that extended to both temporal lobes. The first electroencephalography (EEG), performed three months after initial consultation, was normal and a second EEG, five months later, revealed generalized slow activity. Then the patient abruptly worsened showing akinetic-rigid Parkinsonism with pseudobulbar syndrome and gait impairment with motor apraxia, visuospatial discoordination and evident frontal dysfunction features such as grasping, palmomental reflex and brisk perioral reflexes. Sensory examination could not be performed at that time. Differential diagnosis included dementia with Lewy bodies, corticobasal ganglionic degeneration, or prion disease. The patient was hospitalized to perform a lumbar puncture; examination of her cerebrospinal fluid showed two cells with normal biochemical and microbiologic parameters including negative 14-3-3 test, serology against Borrelia burgdorferi and Rapid Plasma Reagin (RPR) test. From then on for the next 25 months the patient was hospitalized at home. She was then taken to hospital due to pneumonia. At that time she was totally dependent with incontinence and an akinetic-mutism-like syndrome. She died two months later. Informed consent was obtained from the patient’s relatives for autopsy.

Necropsy was performed within 24 hours after death and samples from nine locations were taken in duplicate for histopathology and molecular processing. Briefly, each sample was fixed in 4% formalin, treated with formic acid and embedded in paraffin. Sections were stained with hematoxylin and eosin or processed for PrP immunohistochemistry with and without PK pretreatment. Frozen non-fixed samples were submitted to immunoblotting for band pattern identification, and comparative enzyme-linked immunosorbent assay (ELISA) for PK-sensitivity analyses were performed as described previously [5].

### Neuropathology

**Figure 1:**
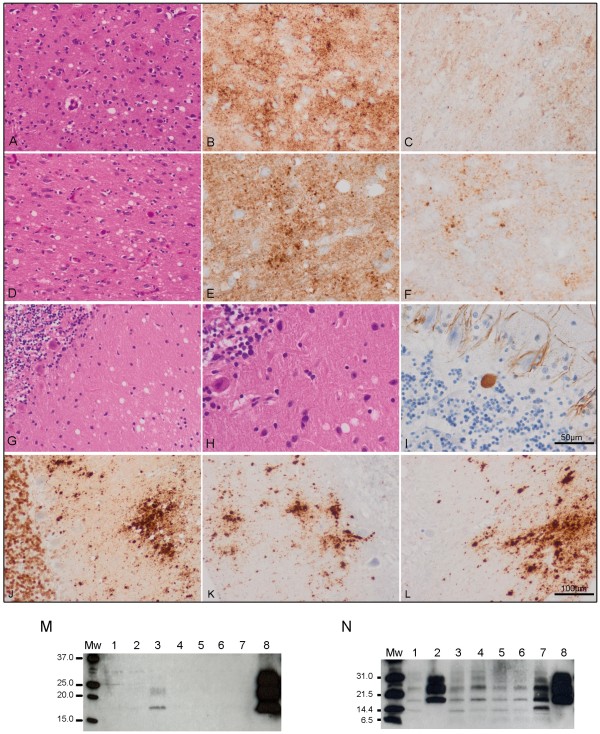
**Immunohistological (A-L) and immunoblotting (M-N) results. AC**: frontal cortex; **DF**: temporal cortex; **GL**: cerebellum. Spongiform change in the frontal (**A**) and temporal (**D**) cortex and molecular layer of the cerebellum (**G**, **H**) is accompanied by moderate neuronal loss in cortex (**A**, **D**) and torpedoes in the granular layer of the cerebellum (**I**). PrP-immunoreactive (PrP-ir) deposits are seen in the cerebral cortex and cerebellum (**B**, **E**, **J**). PrP-ir is largely reduced in the cerebral cortex after proteinase K (PK) treatment, except for small PrP-ir dots following a dot-like or target-like pattern (**C**, **F**). By contrast, PrP-ir in the molecular layer of the cerebellum, in the form of elongated plaque-like deposits, is preserved after PK treatment (**K**, **L**); PrP plaques in the granular layer are absent. Paraffin sections: **A**, **D**, **G**, **H**: hematoxylin and eosin staining; I: phosphorylated neurofilament immunohistochemistry; **B**, **C**, **E**, **F**, **JL**: PrP immunostaining (3F4 antibody) without (**B**, **E**, **J**) and with (**C**, **F**, **K**, **L**) PK treatment. **A**, **D**, **G**, **J**, **K**, **L**: × 200 (bar in L, 100μm); **B**, **C**, **E**, **F**, **H**, **I**: × 400 (bar in I, 50μm). PK was used according to the indications of the supplier: 1 drop of PK concentrate (DAKO, S2019) in 1.6mL of DAKO ChemMate TM PK diluent (S2032) for 15 minutes. **M**: Routine immunoblotting conditions (10% brain homogenate and final PK concentration of 440μg/mL) as described elsewhere [5] and five minutes of film exposure time. PK pretreated brain regions corresponded to occipital cortex (lane 1), putamen/globus pallidus (lane 2), cerebellum (lane 3), parietal cortex (lane 4), thalamus (lane 5), frontal cortex (lane 6), temporal cortex (lane 7), sporadic Creutzfeldt–Jakob disease (sCJD) VV2 reference case occipital cortex (lane 8). **N**: Immunoblotting of PK pretreated samples with less stringent conditions (TeSeE® Western Blot Kit, Bio-Rad) and detection with 3F4 antibody (Dako, dilution 1:3000) as previously described [5] at ten minutes film exposure time. Brain regions corresponded to occipital cortex (lane 1), cerebellum (lane 2), parietal cortex (lane 3), thalamus (lane 4), frontal cortex (lane 5), temporal cortex (lane 6), variably protease-sensitive prionopathy 129MV parietal cortex (lane 7) [5] and sCJD VV2 reference case frontal cortex (lane 8). Molecular weight standards are indicated in kDa: (**M**) SDS-PAGE Standards, broad range, Bio-Rad and (**N**) Precision Plus Protein Unstained Standards, Bio-Rad.

Spongiform change was found in the upper (Figure 1A) and inner (Figure 1D) layers of the cerebral cortex, including frontal and temporal cortex, entorhinal cortex and subiculum. The striatum and thalamus showed moderate spongiform change. PrP immunoreactivity followed a synaptic pattern and was accompanied by dot-like (target-like according to the nomenclature of Zou *et al*. [6]) deposits, particularly in the deep cortical layers. PrP deposition was largely sensitive to PK treatment, except for the majority of target-like deposits that were PK-resistant (Figure 1B, 1C, 1E, 1F). Perineuronal PrP-immunoreactive deposition was absent.

The cerebellum showed spongiform change in the molecular layer, moderate loss of Purkinje cells and occasional axonal torpedoes (Figure 1G–I). PrP immunohistochemistry showed elongated plaque-like deposits in the molecular layer which were resistant to PK pretreatment (Figure 1J–L). Plaque-like deposits in the granular layer of the cerebellum as are often seen in sCJD VV2 were absent.

Accompanying findings were neurofibrillary tangles in the entorhinal cortex, subiculum, hippocampus, inferior temporal cortex, and rarely in the frontal cortex, and β-amyloid plaques, diffuse and neuritic, in the cerebral cortex corresponding to Alzheimer’s disease-related changes in neuritic stages VB of Braak.

### *PRNP* analysis

The entire PRNP open reading frame was amplified by polymerase chain reaction (PCR ) using genomic DNA extracted from fresh brain tissue. PCR was carried out in a final volume of 25μl with 45ng DNA, 0.1mM dNTP, 1mM MgCl2, 0.2μM of each primer (Forward: 5′-CCATTGCTATGCACTCATTC-3′; Reverse: 5′-CGGGACAAAGAGAGAAGAAA-3′), 0.2mg/ml bovine serum albumin BSA, 0.5 units of Taq Polymerase and 1x Taq buffer, under the following conditions: initial denaturation step at 95°C for ten minutes, 35 cycles of denaturation at 95°C for 45 seconds, annealing at 60°C for 30 seconds and elongation at 72°C for one minute, and a final elongation step at 72°C for ten minutes. Sequence analysis was done directly by automated sequencing. The patient was homozygous for valine at codon 129 of *PRNP* and no mutations, insertions or silent polymorphisms were found.

### Biochemistry and western blotting

Routine immunoblot examinations [5] revealed lack of signal in all brain regions and a weak three band pattern of pathogenic PrP in the cerebellum corresponding to a 2A protein type (Figure 1M). The recent description of a case of protease-sensitive prionopathy 129MV and the use of technologies based on PrP detection without proteinase K treatment [5] allowed us to reanalyze this case. Immunoblotting under less restrictive conditions [5] and incubation with 3F4 antibody revealed a ladder-like PrP pattern in all brain regions excluding the cerebellum, which maintained the three band pattern typical of sCJD type 2A (Figure 1N). The evaluation of PrP PK sensitivity in the comparative ELISA showed a PrP mean signal detection rate decreased by 71% [5]. The highest values ranged from 72% to 78% for cortex, striatum and thalamus, and the lowest value of 43% corresponded to the cerebellum. This could partially explain the absence of protein signal in immunoblot in the aforementioned brain regions and the weak signal detected in the cerebellum.

## Conclusion

The first 129VV protease-sensitive prionopathy series reported [1] and further characterized (PSPr or VPSPr) [2,4,6] presented with neurobehavioral and psychiatric signs such as behavior and mood changes, speech deficit and cognitive impairment, followed by progressive motor decline and dementia. We describe the first Spanish patient with findings comparable to the cases previously described [1,2,4,6]. Furthermore, molecular typing of PrP in different regions of the encephalon (excepting cerebellum) showed a ladder-like pattern ranging from 29 to 7kDa similar to that found in the 129VV PSPr cases [1,2,4,6]. However, a striking observation of this case was that PrP type in the cerebellum was reminiscent of sCJD type VV2 because PrP-resistant elongated deposits were present in the molecular layer plaques but absent in the granular layer. Reanalysis of different brain homogenates confirmed this finding. Co-occurrence of different PK-sensitivity PrP types in the same PSPr patient has, to the best of our knowledge, not yet been reported [1,2,4,6], even though coexistence of different classical PK-resistant PrP forms is a common finding in other prion diseases [7-15]. This observation constitutes a novel finding which broadens the spectrum of molecular and clinicopathological features of spongiform encephalopathies.

## Consent

Written informed consent was obtained from the patient's next-of-kin for publication of this case report and any accompanying images. A copy of the written consent is available for review by the Editor-in-Chief of this journal.

## Competing interests

The authors declare that they have no competing interests.

## Authors’ contributions

AR carried out the molecular and genetic analysis. JA organized and managed the necropsy and the epidemiological information. AL, IF, NV and BA collected and evaluated clinical and pathological information and samples. AR, AL, IF, RJ and JZ participated in the drafting of the manuscript and critically discussed and wrote specific parts of it. AR, RJ and JG conceived the study. All authors read and approved the final manuscript.
